# CONSORT compliance in randomized surgical trials assessing the analgesic and anti-inflammatory effectiveness of preoperative drug management of patients undergoing mandibular third molar surgery: a scoping review

**DOI:** 10.4317/medoral.25494

**Published:** 2022-09-29

**Authors:** Edson Luiz Cetira-Filho, Alessandra Fragoso Vieira, Pedro Henrique da Hora Sales, Paulo Goberlânio de Barros Silva, Fábio Wildson Gurgel Costa

**Affiliations:** 1 DDS,OMFS, MSc, PhD student. Division of Oral and Maxillofacial Surgery, Postgraduate Program in Dentistry, Federal University of Ceará, Fortaleza, Ceará, Brazil. Professor at UNICHRISTUS, Fortaleza, Ceará, Brazil; 2 DDS, MSc student. Division of Oral Radiology, Postgraduate Program in Dentistry, Federal University of Ceará, Fortaleza, Ceará, Brazil; 3 DDS,OMFS, MSc, PhD student. Division of Prosthesis and Oral and Maxillofacial Surgery, Dental School, Federal University of Pernambuco, Recife, Pernambuco, Brazil; 4 DDS, OP, MSc, PhD. Division of Oral Pathology, Dental School, Christus University (Unichristus), Fortaleza, Ceará, Brazil; 5 DDS, OMFS, MSc, PhD. Division of Oral and Maxillofacial Surgery, Postgraduate program in Dentistry, Federal University of Ceará, Fortaleza, Ceará, Brazil

## Abstract

**Background:**

Investigate methodological quality of clinical trials in mandibular third molar surgery and its compliance with the consort statement.

**Material and Methods:**

An electronic search was performed in five journal websites, chose the five scientific journals with the greatest impact factor in oral and maxillofacial surgery according to the SCImago Journal Rank. The compliance of studies with the CONSORT statement was assessed. Also, the risk of bias of each study was evaluated.

**Results:**

Twenty-nine studies were included. The average CONSORT compliance score was 25.50 (79.68%). Most studies were performed in the Americas (n = 14, 48.3%) and Asia (n = 10, 34.5%). Parallel-group (n=15, 51.7%) and split-mouth RCTs (n=11, 38%) were the most prevalent study design. An inverse correlation was observed between the year of publication and the number of Scopus citations (*p*<0.001), time between acceptance and publication (*p*<0.001), and time between study completion and publication (*p*=0.040).

**Conclusions:**

Understanding the correct use of guidelines, such as the CONSORT statement, is necessary to reduce methodological errors and possible bias, thereby ensuring reliable knowledge dissemination.

** Key words:**CONSORT, clinical trial, mandibular third molar, oral surgery, quality assessment tool.

## Introduction

Removal of impacted mandibular third molars is a routine surgical procedure in the dental clinic, especially for oral and maxillofacial surgeons (1). This procedure often requires soft tissue flaps and bone tissue removal to access the tooth; therefore, an inflammatory reaction with moderate to severe pain, edema, and trismus is generally expected after extraction (2). These innate bodily responses may result in significant discomfort to the patient, limiting their daily activities and consequently decreasing their quality of life (1,3,4).

In this context, measures aiming at minimizing these negative effects after impacted lower third molar surgery are necessary. Choosing an adequate surgical technique, optimization of surgical time, and adequate analgesic regimen could not only reduce the need for extra consultations during the postoperative follow-up but also decrease the patient's anxiety regarding the treatment, thereby ensuring a more comforTable postsurgical recovery (5,6,7).

Several studies evaluating the preemptive effect of analgesic and non-steroidal anti-inflammatory drugs in controlling pain, edema, and trismus after removal of lower third molars have been published (1,4-6,8-10). Randomized clinical trials (RCTs) and systematic reviews of RCTs with or without meta-analysis are investigations capable of providing a high degree of scientific evidence for health care interventions. Therefore, these types of studies are the best source of research to compare the effectiveness of different drug intervention protocols. However, RCTs must be well designed and follow rigorous methodological standards to produce robust scientific evidence; otherwise, they could generate biased results, possibly leading to an under-or overestimation of the effect of an intervention (11,12).

The CONSORT statement is a list of recommendations developed to improve the quality and standardization of RCT reporting. After its publication in 1996, the checklist was revised in 2001 and 2010 (13). These guidelines are of great relevance for reporting non-inferiority/equivalence and parallel-group studies as well as factorial, cluster, and crossover trials. The list of recommendations currently comprises 25 essential items in addition to a flow diagram to assist in documenting the flow of research participants throughout the trial. Thus, the study becomes more easily understandable and reproducible, while also offering greater certainty of evidence, as it follows a strict methodology (14).

However, the incorporation of the recommendations provided in each item has been considered challenging by some authors. Hopewell *et al*. (2010) (15) observed that a high percentage of CONSORT items had not been properly cited among RCTs indexed in PubMed, such as allocation concealment (75%), flow diagram (72%), sample size calculation (55%), primary outcome (47%), and blinding (44%). The study by Siddiq *et al*. (2019) (16) evaluated the adherence to CONSORT in 369 RCTs published between 2011 and 2016 in periodontology journals and found that less than 40% of the articles adhered to the methodology, results, and discussion items, and none of the trials fully complied with the guidelines. Furthermore, the investigation by Reis *et al*. (2018) (13) evaluating the adherence to CONSORT and the risk of RCT bias on adhesive systems in non-carious cervical lesions revealed that information on items such as flow diagram, blinding, and sample size calculation was lacking in nearly 80% of the included articles, highlighting the importance of RCT reporting quality studies in other areas of dentistry such as oral and maxillofacial surgery.

Therefore, the purpose of this scoping review was to assess the adherence to the CONSORT statement by RCTs evaluating the effectiveness of preoperative administration of analgesic and anti-inflammatory drugs on inflammatory outcomes resulting from mandibular third molar surgery.

## Material and Methods

- Protocol and registration

This review was carried out according to the Preferred Reporting Items for Systematic Reviews and Meta-analyses extension for Scoping Reviews (PRISMA-ScR) (17) Checklist and followed the methodology for scoping reviews of the Joanna Briggs Institute (JBI) Manual for Evidence Synthesis.

The review question was: “Do randomized clinical trials in oral surgery comply with the CONSORT statement?”

The primary outcome of this scoping review was to assess how clinical trials in oral surgery (lower third molar removal) were conducted and whether they complied with the CONSORT statement. In addition, the secondary outcome was to determine whether there is an association between the presence of risk of bias and compliance with the CONSORT guidelines.

- Search information and search strategy

Appropriate truncations and word combinations were selected and adapted for each scientific journal website. We chose the five scientific journals with the greatest impact factor in oral and maxillofacial surgery according to the SCImago Journal Rank (SJR) available on the SCImago website (https://www.scimagojr.com/journalrank.php?category=3504).

- Inclusion criteria

We included only randomized controlled trials, regardless of their design (parallel-group, cluster, or crossover) or main outcome, in which participants underwent mandibular third molar surgery. We did not apply any restrictions on participant age or tooth position.

- Exclusion criteria

The following studies were excluded: RCTs published before 1996 (pre-CONSORT period); studies not written in languages using the Latin (Roman) script; and conference abstracts, letters, literature reviews, case reports, clinical observations, author's personal opinions, book chapters, and editorials.

- Information sources

Detailed individual search strategies for each of the following scientific journal websites were performed: International Journal of Oral and Maxillofacial Surgery (IJOMS), Journal of Cranio-Maxillo-Facial Surgery, British Journal of Oral and Maxillofacial Surgery, Journal of Oral and Maxillofacial Surgery (JOMS), Oral Surgery, Oral Medicine, Oral Pathology and Oral Radiology (OOOO). The search included all articles published on or before October 10, 2021, with no time restrictions.

The terms (MeSH terms and free keywords) used for the initial search were: (“Third Molar” OR “Third Molars” OR “Wisdom Tooth” OR “Wisdom Teeth”) AND (“randomized controlled trial” OR “controlled clinical trial” OR “clinical trial” OR trial).

- Study selection

The selection process was completed in two phases. In phase 1, two reviewers (ELCF and PHHS) independently screened the titles and abstracts of all electronic database citations. Phase 1 was performed using a web application for systematic reviews (Rayyan®, Qatar Computing Research Institute, Doha, Qatar). Articles that did not appear to meet the inclusion criteria were excluded. In phase 2, the same reviewers independently applied the inclusion criteria to the full text of the articles.

- Data collection process

One author (ELCF) extracted data from the selected studies. A second author (PHHS) cross-checked all the obtained information. Any disagreements between the two authors were discussed until its complete resolution. A third author (FWGC) made the final decision whenever the two authors failed to reach an agreement.

- Data items

The following information was recorded from the selected studies: authors and publication year; journal of publication; methodological design; journal metrics; country of origin of the study; sample size; outcomes investigated in the trial; follow-up period; source of funding; registration of the clinical trial; and outcomes of interest for this scoping review.

- Compliance with the CONSORT statement

The compliance of studies with the CONSORT statement was assessed using a CONSORT compliance evaluation tool (13). This instrument consists of 16 items extracted from the topics of the methods and results sections of the 2010 CONSORT statement (13).

The score per item ranged from 0 to 2 as follows: 0 = no description, 1 = poor description, and 2 = adequate description. Each item received equal weight. Trials with adequate descriptions (score 2) on all CONSORT items received a maximum score of 32.

Two authors (E.L.C.F. and A.F.V.) scored the studies using the CONSORT compliance evaluation tool. In case of doubts concerning the interpretation of the tool items, a third author (F.W.G.C.) was contacted for discussion and final decision. Evaluators were not blinded to the study authors.

- Risk of bias in individual studies

The risk of bias was assessed independently by two review authors (E.L.C.F. and P.H.H.S.). Any disagreement between them over the risk of bias items was resolved through discussion with a third reviewer (F.W.G.C.). The JBI Critical Appraisal Checklist for Randomized Controlled Trials was adopted and used.

The Meta-Analysis of Statistics Assessment and Review Instrument (MAStARI) was used to assess the risk of bias (RoB) of the included studies. The RoB was based on studies with similar methodologies, ranging from ‘high’ (when the study had a ‘yes’ score of less than 49%) to ‘moderate’ (50-69%), and ‘low’ (70% or more) (Haas *et al*., 2016) (18). RevMan Software (Review Manager, version 5.3, Cochrane Collaboration, Copenhagen, Denmark) was used to generate the RoB summary adapted for the MAStARI tool questions.

- Statistical analysis

Data were expressed as absolute frequency and percentage or mean and standard deviation values and compared using Spearman's correlation and Mann-Whitney or Kruskal-Wallis/Dunn tests (non-parametric data).

## Results

**Figure 1:**
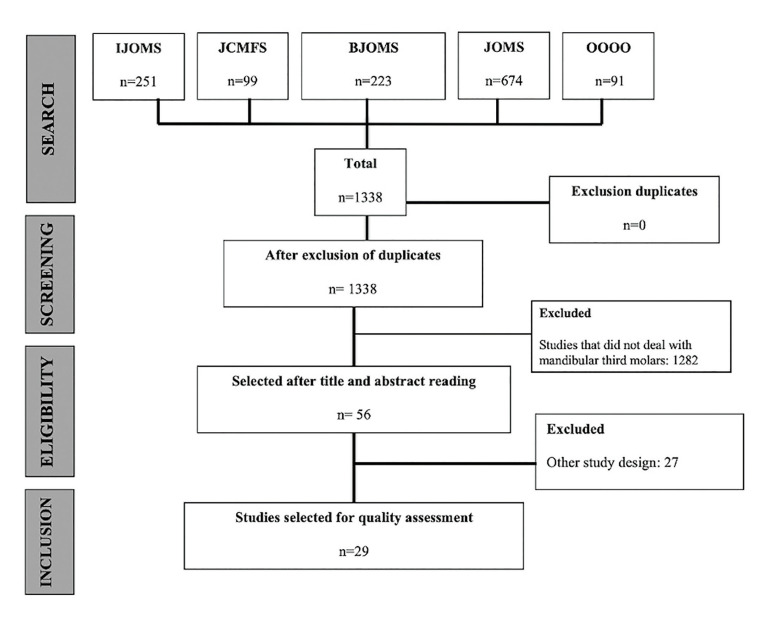
Flowchart of the study selection process.

A search algorithm was applied to five databases of oral and maxillofacial surgery journals chosen by the latest SJR index. The electronic search retrieved a total of 1,338 records. After title and abstract screening, 29 RCTs were selected (Fig. 1).

Table 1 displays the characteristics of the included studies. Most studies were performed in the Americas (n = 14, 48.3%) and Asia (n = 10, 34.5%). Parallel group (n=15, 51.7%) and split-mouth RCTs (n=11, 38%) were the most prevalent study design.

There was a direct correlation between the JCR index and the year of publication (*p*=0.009). An inverse correlation was observed between the year of publication and the number of Scopus citations (*p*<0.001), time between acceptance and publication (*p*<0.001), and time between study completion and publication (*p*=0.040). The number of authors was directly correlated with the year of publication (*p*=0.032) as well as the overall CONSORT compliance (*p*=0.001) and the methodology compliance scores (*p*=0.009). The number of participating centers was inversely correlated with the JCR index (Table 2, Table 3).

**Table 1:**
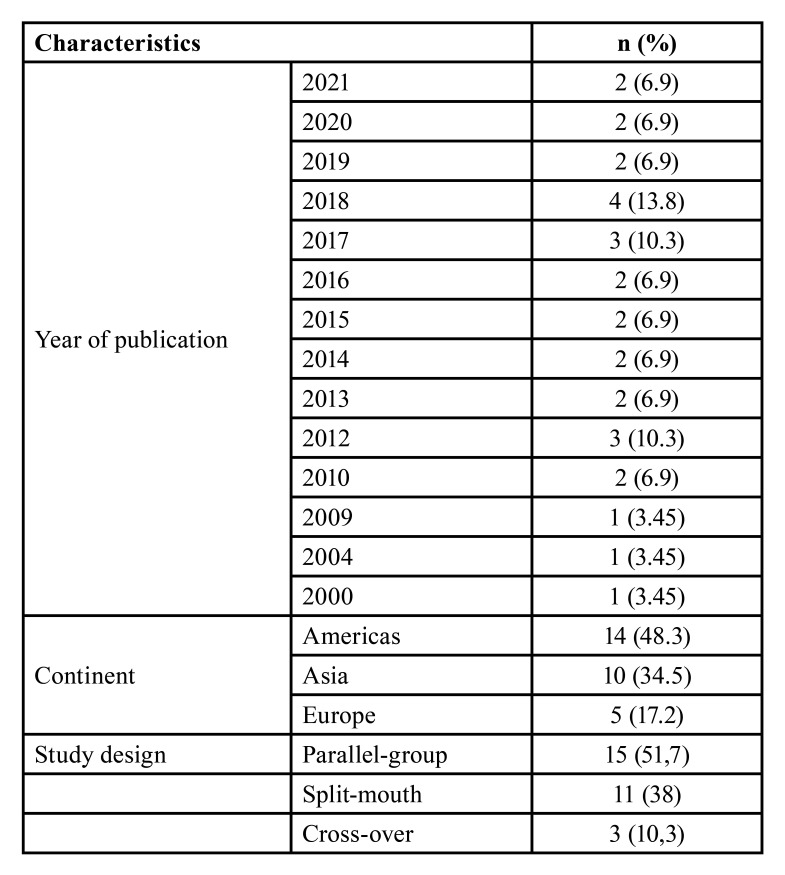
Characteristics of included studies.

**Table 2:**
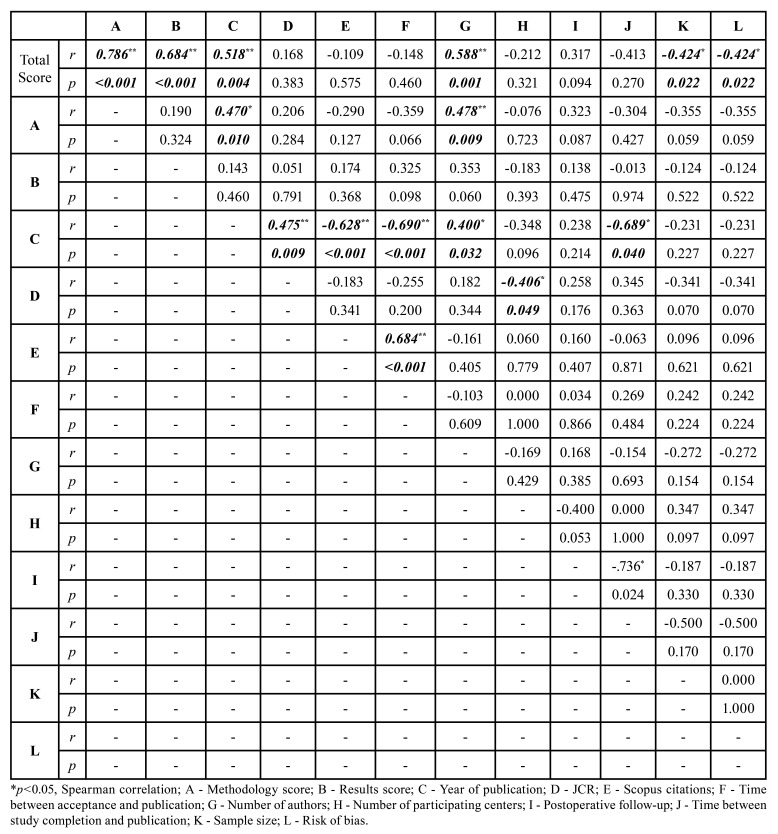
Methodology design.

**Table 3:**
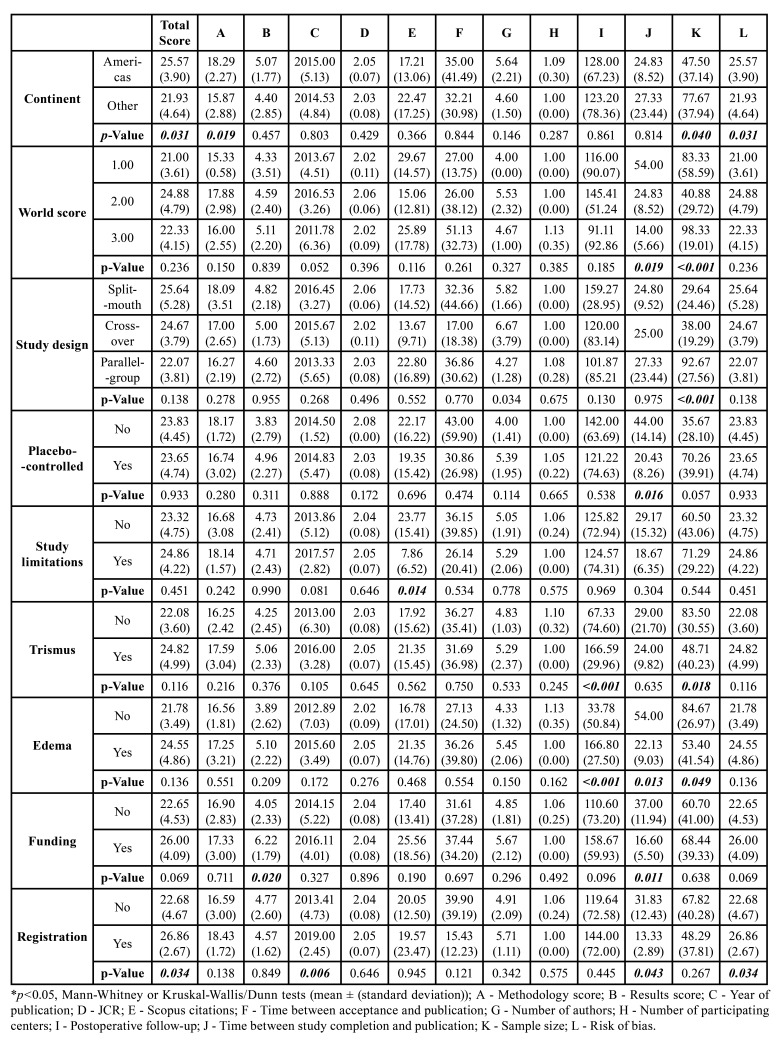
Correlation of the mean scores with main results.

Studies from the American continent had higher overall CONSORT scores (*p*=0.031), higher scores in methodology (*p*=0.019), and a larger sample size (*p*=0.040) than studies from other locations. Parallel group studies had a higher mean number of participants (*p*<0.001), whereas placebo-controlled studies had a shorter time between study completion and publication (*p*=0.016). Moreover, studies that did not mention limitations had more Scopus citations (*p*=0.014).

Trismus was the only inflammatory outcome evaluated in all studies. Furthermore, all papers were from journals that encourage compliance with the CONSORT statement and require research ethics committee approval. Trismus evaluation was directly associated with longer postoperative follow-up (*p*<0.001) and inversely associated with sample size (*p*=0.018). Edema evaluation was directly associated with longer postoperative follow-up (*p*<0.001) and time between study completion and publication (*p*=0.013) and inversely associated with sample size (*p*=0.049). Having a funding source reduced the mean time between study completion and publication (*p*=0.011) and was directly associated with higher CONSORT methodology scores (*p*=0.020). Registered trials exhibited higher overall CONSORT scores (*p*=0.034) and were more frequently reported in more recent studies (*p*=0.006).

**Figure 2:**
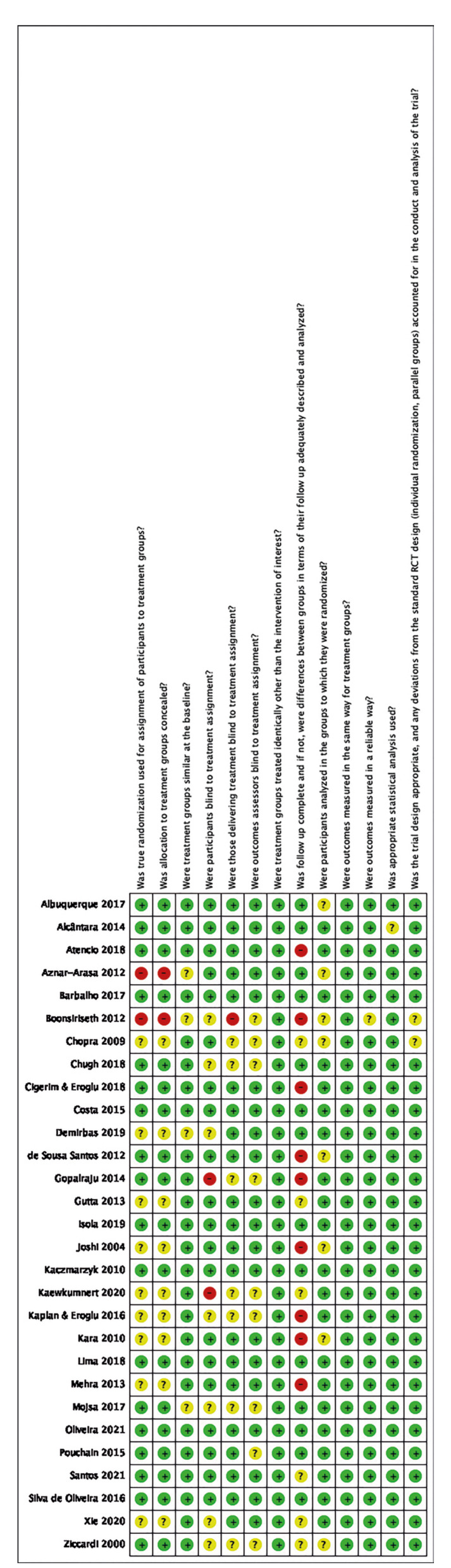
Risk of bias using the JBI tool.

The risk of bias was inversely correlated with CONSORT scores (*p*<0.05) (Fig. 2).

## Discussion

This scoping review aimed to determine the reporting quality of randomized clinical trials in oral and maxillofacial surgery studies following the CONSORT statement. The results of this study evidenced that the form of reporting of some trials omit important information that may compromise the research transparency and reproducibility, such as randomization, allocation, and blinding. Consequently, the scientific evidence generated is negatively affected, which may render internal and external validity inadequate.

The first study involving third molar surgery dates from 1945 (19). The first study characterized as a clinical trial was published in 1973 (20), whereas the first study characterized as a randomized clinical trial was published in 1978 (21). This fact highlights that despite decades of study in dentistry, this topic is still considered relatively new, which affected the number of articles selected in this review.

The randomized clinical trials included in this scoping review evaluated the main clinical outcomes associated with third molar surgery, such as pain, edema, and trismus. Other methodological variables such as the length of clinical follow-up have been also cited, albeit this could reflect the study design (parallel-group, split-mouth, or cross-over), randomization method, or whether the study was placebo-controlled or not.

The quality of the selected clinical trials was assessed using a CONSORT compliance evaluation tool, which had already been performed in previous studies in the field of restorative dentistry (13). Items related to the title and abstract, introduction, and discussion of the studies were not evaluated because compliance with these items would not impact the quality or affect the risk of bias of the studies. The data from the items of the CONSORT evaluation tool are described in detail in Table 4, identifying characteristics that should be described or included in future clinical trials.

The compliance of the studies with the CONSORT checklist for the description of the intervention, study design, and number of participants was adequate, obtaining the following compliance values, respectively: 72.4%; 85.17%, and 59.05%. This is an important aspect, as it allows for the reproducibility of surgical techniques with fewer postoperative inflammatory changes after third molar surgery. The items evaluating the description of the number of patients analyzed, eligibility criteria, baseline data, losses and exclusions also presented adequate descriptions, which reduces biases that could generate distortions in the analysis of the results when the follow-up is not correctly performed, or if losses and exclusions are removed. This information must be clear and demonstrate that the analyses were performed with the intent to treat the patient, and the study must describe how the missing data were included in the overall analysis.

**Table 4:**
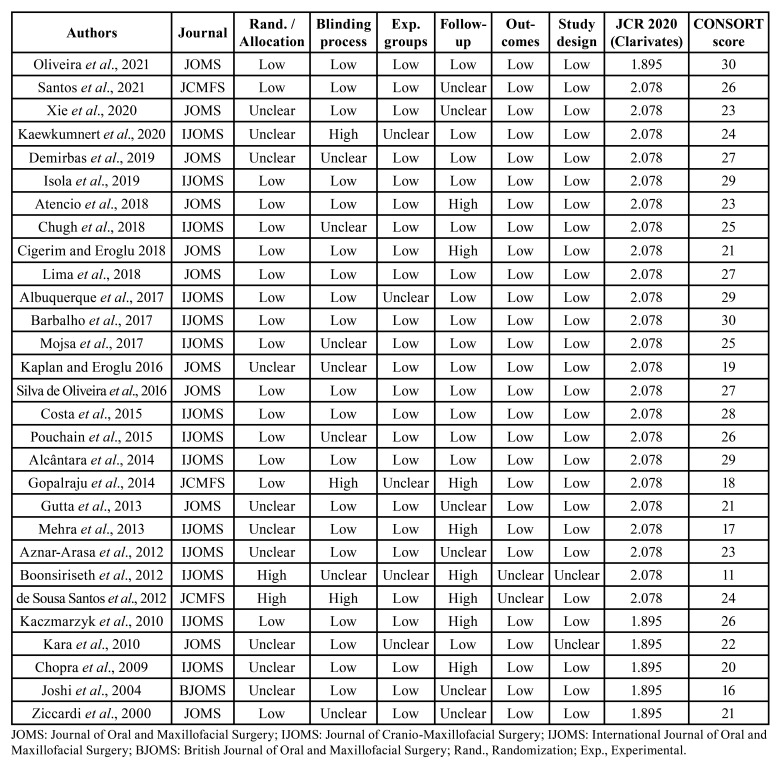
Description of the JBI-related risk of bias and CONSORT scores.

On the other hand, the compliance with items such as registration and protocol of randomized clinical trials was low, with a compliance value of only 24.13%. This might reflect inadequate research planning, questioning whether randomization was correctly performed, as it could result in publication bias, especially in randomized clinical trials with small groups of patients.

The results of this study demonstrated a direct correlation between the number of authors and the overall CONSORT score, which may indicate the need for more authors with clinical, surgical, and research experience. In addition to favoring greater methodological robustness, this step could help ensure blinding, allocation, correct outcome measurement, among other variables, reducing the risk of bias in the study (22).

Furthermore, multicenter studies exhibited higher JCR indices, directly affecting publication time. However, this factor was not favorable for Scopus citations.

Studies from the American continents obtained higher CONSORT scores, indicating that rigorous methodology conducts were followed by the research teams (6,8-10,23-31).

Another important finding was that placebo-controlled studies allowed for a shorter peer-review process before publication, which indicates that this type of design contributes to greater scientific robustness of the research, as observed in the papers authored by Albuquerque *et al*. (2017) (1) and Simone *et al*. (2013) (32).

The studies that evaluated trismus were published in journals that encouraged the use of the CONSORT statement and required research ethics committee approval. These studies were also in direct correlation with longer postoperative follow-ups and inversely correlated with sample size, possibly related to challenges inherent in a cross-sectional study design, in which patients often do not return for long-term clinical evaluations, as reported in the studies by Xie *et al*. (2020) (3); Demirbas *et al*. (2019) (2); Attencio *et al*. (2018) (24); Lima *et al*. (2018) (25); Gopalraju *et al*. (2014) (33); Gutta *et al*. (2013) (28); Kaczmarzyk *et al*. (2010) (34); Joshi *et al*. (2004) (35) and Ziccardi *et al*. (2000) (31). The studies evaluating edema had a longer postoperative follow-up, were published in less time, and, similarly to studies evaluating trismus, an inverse correlation with sample size was observed, also related to the difficulty of patient return visits during the postoperative follow-up.

However, in more recent RCTs, positive/favorable correlations between the year of publication and the risk of bias have been observed, suggesting that researchers are growing increasingly attentive to positive clinical research practices, especially considering the new recommendations for clinical research in oral and maxillofacial surgery, which aim at the establishment of clinical protocols.

The moderately-low number of articles found, despite the comprehensive literature search carried out to locate as many published papers as possible, impaired a more robust statistical analysis of the data and was, therefore, a limitation of this study. This might reflect the difficulty of generating clinical trial protocols including only studies from high impact factor journals (according to the latest SJR index) in the field of oral and maxillofacial surgery.

The manner of reporting the data in clinical trials is an aspect of great relevance to its publication process in high-impact factor journals. Thus, inadequate reporting can have negative consequences for all parties involved, such as the research team, the volunteers, and the scientific community in a global context, including waste of resources and possible negative implications for health decisions regarding third molar surgery. The RCTs with better reporting quality were mostly observed in studies from the American continents, and the total score was significantly associated with bibliometric indexes. Understanding the correct use of guidelines, such as the CONSORT statement, is necessary to reduce methodological errors and possible bias, thereby ensuring reliable knowledge dissemination.
